# Educational

**Published:** 1869-11

**Authors:** 


### EDUCATIONAL.

We have just received the Fourth Annual Announcement of the New York College of Dentistry, by which we learn that its session begins on the 15th inst.

There was, for the past five months, some serious embarrasment resting on this institution, which we are happy to know has been removed, and that it now enters upon its work with renewed energy. We have always hoped and expected large things of this college, and was very sorry, indeed, to hear of its recent troubles; but, perhaps, it was just such a trial as is liable to, and so frequently does come, upon young institutions, to try the resources and test the strength of the enterprise. We trust that in the future there may be no interruption in its course of usefulness.

